# The association of plasma connective tissue growth factor levels with left ventricular diastolic dysfunction in patients with overt hyperthyroidism

**DOI:** 10.3389/fendo.2024.1333001

**Published:** 2024-02-05

**Authors:** Huan Li, Yahui Ren, Linfang Wang, Yuming Li

**Affiliations:** ^1^Department of Ultrasound Medicine, Union Hospital, Tongji Medical College, Huazhong University of Science and Technology, Wuhan, China; ^2^Hubei Province Key Laboratory of Molecular Imaging, Wuhan, China; ^3^Department of Pediatric, Union Hospital, Tongji Medical College, Huazhong University of Science and Technology, Wuhan, China; ^4^Department of Gastrointestinal Surgery, Union Hospital, Tongji Medical College, Huazhong University of Science and Technology, Wuhan, China; ^5^Department of Endocrinology, Union Hospital, Tongji Medical College, Huazhong University of Science and Technology, Wuhan, China; ^6^Hubei Provincial Clinical Research Center for Diabetes and Metabolic Disorders, Wuhan, China

**Keywords:** left ventricular diastolic dysfunction, CTGF, hyperthyroidism, thyroid hormone, echocardiography

## Abstract

**Background:**

Left ventricular (LV) diastolic dysfunction is an independent predictor of future cardiovascular events. Early detection of patients with LV diastolic dysfunction can improve clinical outcomes through active management. However, the assessment of diastolic function is very complicated, and there are currently lack of effective biomarkers to assess the risk of LV diastolic dysfunction. Connective tissue growth factor (CTGF) plays a significant role in cardiac remodeling and dysfunction. We aimed to investigate the associations between plasma CTGF level and the risk of LV diastolic dysfunction in this study and judge its effectiveness in diagnosing LV diastolic dysfunction.

**Methods:**

A total of 169 patients with overt hyperthyroidism were included. LV diastolic function was evaluated and the subjects were divided into normal LV diastolic function group and LV diastolic dysfunction group. Routine clinical medical data, biochemical data, thyroid related parameters and echocardiographic parameters were recorded for analysis.

**Results:**

Compared with normal LV diastolic function group, the LV diastolic dysfunction group had higher age and BMI, as well as lower heart rate, lower serum albumin, lower eGFR, higher serum TgAb and BNP level, and the incidences of hypertension were also higher (all *P* <0.05). Circulating plasma CTGF levels in the LV diastolic dysfunction group were significantly higher (normal LV diastolic function group: 7.026 [5.567-8.895], LV diastolic dysfunction group: 8.290 [7.054-9.225] ng/ml, median [(Interquartile range)], *P* = 0.004); Compared with the lowest quartile group, the crude odds ratios (OR) of LV diastolic dysfunction in the second, third, and fourth quartile group were 3.207, 5.032 and 4.554, respectively (all *P*<0.05). After adjustment for the potentially confounding variables, the adjusted OR values of the third and fourth quartile group had no obvious change. The results of ROC showed that the plasma CTGF had the largest area under the ROC curve, and the value was 0.659 (*P* = 0.005).

**Conclusion:**

The level of circulating plasma CTGF in the LV diastolic dysfunction group was significantly increased. Plasma CTGF level is an independent risk factor for LV diastolic dysfunction. Compared with serum BNP level, the plasma CTGF level may have auxiliary diagnostic value for LV diastolic dysfunction in hyperthyroid patients.

## Introduction

Left ventricular (LV) diastolic dysfunction is an independent predictor of future cardiovascular events, including stroke, atrial fibrillation, congestive heart failure and death (1–4). Our previous study indicated that LV diastolic dysfunction was very common in patients with overt hyperthyroidism, and the prevalence of LV diastolic dysfunction was 35.1% among hyperthyroid patients and significantly higher than the control subjects (5).However, the assessment of LV diastolic function is complex. Cardiac catheterization is the most accurate way to assess LV diastolic function, while it is invasive and cannot be universally used in clinical practice (6). The parameters measured by echocardiography are mainly used to evaluate LV diastolic function at present, and there are multiple parameters used to evaluate LV diastolic function, but their sensitivity and specificity are different (7). Therefore, it is necessary to explore effective biomarkers of LV diastolic dysfunction.

Connective tissue growth factor (CTGF), a secreted extracellular protein, is a member of CCN family (acronym of Cyr61/CEF-10, CTGF/Fisp-12, Nov), which was first discovered in the conditioned medium of human umbilical vein endothelial cells by BRADHAM et al. in 1991 (8–10). Studies have shown that CTGF was a type of pro-fibrotic factor. Dysregulation of CTGF expression has been found to play an important role in inflammation, injury response, wound repair, and fibrosis-related diseases (11, 12). Recently, there is growing evidence that CTGF is a risk factor for heart diseases (13–18). The population studies showed that plasma CTGF was a novel potential biomarker of cardiac dysfunction in patients with chronic heart failure and high levels of CTGF can predict future myocardial infarction (MI) and cardiovascular death in patients with type 2 diabetes (14, 19). *In vitro*, Exogenous CTGF stimulation can not only lead to cardiomyocyte hypertrophy, but also promote the proliferation of cardiac fibroblasts and the deposition of extracellular matrix (8, 20); *In vivo*, studies have shown that CTGF expression of cardiac tissue was upregulated in animal models of hypertrophic cardiomyopathy and MI (21, 22), while cardiac fibrosis and myocardial hypertrophy are important pathogenesis of LV diastolic dysfunction, and there are few studies investigating the association between plasma CTGF level and LV diastolic dysfunction.

Previous studies have shown that a wide range of factors can regulate CTGF expression, such as transforming growth factor β, angiotensin II, aldosterone, endothelin-1, high glucose, free fatty acids and so on (8, 20, 23–25), and CTGF expression levels in tissues and cells begin to increase early in mechanical stimulation or stress loading, and the degree of increase is parallel to the level of stress (26). It was well known that excessive thyroid hormones can activate the synthesis of some cardiac structural and functional proteins, leading to myocardial hypertrophy, increased left ventricular mass and a series of hemodynamic changes (27). Additionally, Hayata et al. showed that CTGF overexpression initially lead to reversible cardiac hypertrophy via activation of Akt pathway, which was followed by irreversible ventricular dilatation for a longer time *in vivo (* 9), which seems to be similar to the effect of thyroid hormone on the heart. Furthermore, in contrast to CTGF, BNP is an antifibrotic factor. Previous studies suggested that serum BNP levels were positively correlated with thyroid hormone levels (28, 29), while it is unclear whether plasma CTGF levels are associated with thyroid hormone levels.

Therefore, the aim of this study was to explore the relationship between the plasma CTGF level and thyroid hormone level in patients with clinical hyperthyroidism, and to explore the correlation between the plasma CTGF level and LV diastolic dysfunction.

## Materials and methods

### Subjects

Overt hyperthyroidism was defined as increased free thyroxine (FT4) and/or free triiodothyronine (FT3) level and a concomitantly suppressed thyroid-stimulating hormone (TSH) level according to the guidelines of the American Thyroid Association Guidelines (30). Besides, it was further confirmed by test of thyroid iodine uptake rate and thyroid imaging. Subjects were newly diagnosed patients with overt hyperthyroidism or had withdrawn anti-thyroid drug for at least 4 weeks. We excluded subjects with tumor, sepsis, severe injury, diabetes mellitus, hyper-lipidaemia, atherosclerosis, chronic renal failure and fibrosis-associated disease, which were associated with an increased circulating CTGF level. In addition, we excluded patients with congenital heart disease or other known heart disease (such as atrial fibrillation or atrial flutter arrhythmias), a history of surgery within 6 months prior to admission, younger than 18 years old, and incomplete clinical data. Finally, a total of 169 patients with overt hyperthyroidism were included in this study between May 2018 and May 2019 in the inpatient department of endocrinology, Union Hospital, Tongji Medical College, Huazhong University of Science and Technology, Wuhan, China. The approval was obtained from the Tongji Medical College of Huazhong University of Science and Technology, and all included subjects signed the informed consent agreement.

### Data collection

The demographic data of included subjects was collected including physical examinations, medical history, history of diseases, laboratory indicators, auxiliary examinations, and medicine use. The estimated glomerular filtration rate (eGFR) was calculated by using the Chronic Kidney Disease Epidemiology Collaboration equation (CKD-EPI) on the basis of serum creatinine (Scr) level (31). Hypertension was defined as systolic blood pressure (SBP)≥140 mmHg or (and) diastolic blood pressure (DBP) ≥90 mmHg at least twice in inpatient, or the use of oral anti-hypertensive medications, or any self-reported history of hypertension. Hyperuricemia was defined as serum uric acid (SUA) concentration >420 mmol/L in male and >360 mmol/L in female.

### Blood sampling and measurement of CTGF level

Venous blood samples were obtained into containing EDTA tubes in the morning of the second day following admission and after at least 8 h overnight fasting for later measurement. The plasma was separated from the blood cells within 2 h of sample collection by centrifugation at 2500 g for 15 min at room temperature. Plasma was separated, frozen and stored at –80°C. Plasma levels of CTGF were determined by a commercial enzyme-linked immunosorbent assay. All procedures were strictly in accordance with the manufacturer’s instructions.

### Transthoracic echocardiography

All the enrolled subjects underwent complete transthoracic echocardiography using echocardiography system with a 1-5 MHz transducer (GE Vivid 7; Vingmed; Philips EPIQ 7C and Philips IE33). The left ventricular end-diastolic diameter (LVEDD), interventricular septum thickness (IVST), left atrium diameter (LAD), right ventricular end-diastolic diameter (RVEDD), right atrium diameter (RAD) and left ventricular ejection fraction (LVEF) were recorded. In addition, the peak velocities of the early (E-wave) (MVE) and late (A-wave) (MVA) phase of the mitral inflow pattern were measured from an apical four-chamber view, and calculated the ratio of MVE/MVA (E/A ratio). The motion spectrum e/a of the basal segment of the interventricular septum was measured using tissue doppler imaging. The definition of LV diastolic dysfunction was diagnosed by two experienced sound-operator according to the mitral E/A ratio or (and) the septal basal regions e/a ratio. The cut off was less than 1, which indicates impaired myocardial relaxation (30).

### Statistical analysis

The shapiro-Wilk test was used to determine whether continuous data follow a normal distribution. Continuous variables with normal distribution were expressed as mean ± standard deviation, while the skewed distribution data were expressed as median (interquartile range). Categorical variables were expressed in frequency (percentage). The normally distributed continuous variables between groups were compared using t test. The Mann-Whitney U test was used to compare continuous variables with skewed distributions. The Chi-square test was used to compare categorical variable. Besides, spearman correlation analysis was used to explore the correlation between plasma CTGF level and cardiac echocardiography parameters and thyroid related parameters. Moreover, patients were divided into four equal groups according to plasma CTGF levels. Logistic regression analysis model was used and confounders were adjusted to explore the correlation between plasma CTGF levels and the incidence of LV diastolic dysfunction. The receiver operating characteristic curves (ROC) were plotted to evaluate the diagnostic efficacy of CTGF, BNP and BNP/CTGF in LV diastolic dysfunction in patients with hyperthyroidism. All tests were two-sided, and P-values <0.05 were considered statistically significant. SPSS 22.0 (SPSS, Chicago, Illinois, USA) was used for statistical analysis.

## Results

### Baseline characteristics of hyperthyroidism patients with and without LV diastolic dysfunction

The characteristics of included subjects are shown in **Table 1**. A total of 169 patients diagnosed with overt hyperthyroidism were included finally, including 118 subjects with normal LV diastolic function and 51 subjects with LV diastolic dysfunction. The median age was 36 years old (interquartile range, 28-49), and 70.4% of them were female. There was no significant difference between two groups in terms of sex ratio, the duration of disease, SBP, DBP, ALT, AST, Scr, BUN, SUA, electrolyte level (sodium, potassium, calcium), FT3, FT4, TPOAb, TRAb, TSH, thyroid weight and incidences of hyperuricemia (*P*>0.05). Compared with subjects with normal LV diastolic function, subjects in the LV diastolic dysfunction group had higher age and BMI, as well as lower heart rate, lower serum albumin, lower eGFR, higher serum TgAb and BNP level, and the incidences of hypertension were also higher (*P*<0.05).

**Table 1:** Baseline characteristics of hyperthyroidism patients with or without LV diastolic dysfunction.

	All (N= 169)	Without LV diastolic dysfunction (N=118)	With LV diastolic dysfunction (N= 51)	*P*-value
Age (years)	36 (28-49)	32 (27-45)	49 (39-57)	<0.001
Female(n/%)	119 (70.4%)	82 (69.5%)	37 (72.5%)	0.689
Duration (years)	2.00 (0.17-6.00)	1.50 (0.17-5.00)	2.00 (0.17-8.00)	0.231
BMI (kg/m^2^)	21.0 ± 2.8	20.5 ± 2.8	22.1 ± 2.6	0.001
SBP (mmHg)	127 ± 14	126 ± 13	130 ± 17	0.083
DBP (mmHg)	77 ± 10	77 ± 10	77 ± 10	0.755
HR (beats/min)	96 (83-105)	99 (85-107)	88 (78-100)	0.012
Comorbidities				
Hypertension	32 (18.7%)	16 (13.6%)	16 (31.4%)	0.007
Hyperuricemia	26 (15.4%)	18 (15.3%)	8 (15.7%)	0.857
ALT (U/L)	31 (19-43)	32 (20-45)	28 (17-40)	0.207
AST (U/L)	24 (19-31)	25 (19-31)	23 (18-29)	0.475
ALB (g/L)	39.5 ± 3.3	40.0 ± 3.1	38.3 ± 3.7	0.002
Scr (mg/dL)	0.47 (0.39-0.56)	0.47 (0.38-0.55)	0.46 (0.41-0.59)	0.469
BUN (mmol/L)	4.64 ± 1.20	4.56 ± 1.14	4.84 ± 1.30	0.161
SUA (umol/L)	303.2 (251.6-358.7)	300.0 (249.7-357.8)	311.0 (265.3-373.0)	0.430
eGFR (ml/min/1.73m^2^)	129.1 ± 16.7	133.4 ± 15.1	119.0 ± 16.0	<0.001
Sodium (mmol/L)	140.9 ± 1.8	140.8 ± 1.6	141.2 ± 2.2	0.329
Potassium(mmol/L)	3.92 (3.70-4.09)	3.92 (3.75-4.09)	3.90 (3.66-4.10)	0.339
Calcium (mmol/L)	2.29 ± 1.31	2.31 ± 1.21	2.27 ± 1.48	0.078
BNP (pg/ml)	66.2 (20.4-105.5)	66.6 (18.8-105.5)	64.7 (25.7-152.0)	<0.001
FT3 (pmol/L)	27.2 (17.0-35.8)	27.9 (18.0-36.1)	25.2 (13.4-35.2)	0.473
FT4 (pmol/L)	66.7 (42.1-100.0)	67.4 (43.9 -100.0)	65.2 (39.6-100.0)	0.483
Undetectable TSH	125 (74.0%)	85 (72.0%)	40 (78.4%)	0.384
TPOAb (IU/ml)	289.7 (114.4-403.8)	285.63 (105.1-365.6)	289.8 (136.2-600.0)	0.369
TgAb (IU/ml)	250.0 (33.8-552.5)	171.7 (20.0-552.5)	456.1 (88.0-552.5)	0.026
TRAb (IU/L)	14.53 (5.92-22.23)	14.95 (6.42-21.88)	12.90 (4.40-26.51)	0.838
Thyroid mass (g)	66.1 (52.1-93.3)	67.9 (52.1-94.0)	64.7 (50.1-93.0)	0.606

HR, heart rate; BMI, body mass index; DBP, diastolic blood pressure; SBP, systolic blood pressure; AST, aspartate aminotransferase; ALB, albumin; ALT, alanine aminotransferase; Scr, Serum creatinine; BUN, blood uric nitrogen; SUA, Serum uric acid; BNP, brain natriuretic peptide; TSH, thyroid stimulating hormone; FT4, free thyroxine; FT3, free triiodotronine; TPOAb, anti-thyroid peroxidase antibody; TgAb, anti-thyroglobulin antibody; TRAb, thyrotropin receptor antibody; eGFR, estimated glomerular filtration rate.

BMI was calculated as weight in kilograms divided by height in meters-squared (kg/m2).

Continuous variables were presented as mean ± SD or median (interquartiles) according to distribution, Categorical variables were presented as number (%).

Differences between groups were analysed by using Student’s t-test, the Mann-Whitney test, or Chi-square test, as appropriate.

### Comparison of echocardiographic parameters between hyperthyroidism patients with or without LV diastolic dysfunction

The comparison of cardiac echocardiography data between the two groups are shown in **Table 2**. Compared with normal diastolic function group, LAD, IVST and MVA were significantly higher in patients with diastolic dysfunction (both *P* < 0.05), while the MVE and E/A ratio were significantly lower in subjects with diastolic dysfunction (both P < 0.001). Aortic regurgitation was observed in 31.4% of patients with LV diastolic dysfunction, which was significantly higher than that in the normal LV diastolic function group. There was no significant difference in incidence of mitral regurgitation, tricuspid regurgitation and pulmonary hypertension.

**Table 2:** Comparison of echocardiographic parameters between hyperthyroidism patients with or without LV diastolic dysfunction.

	Without LV diastolic dysfunction (N=118)	With LV diastolic dysfunction (N= 51)	*P* -value
LAD (cm)	3.3 (3.0-3.5)	3.5 (3.2-3.9)	<0.001
LVEDD (cm)	4.6 (4.3-4.8)	4.5 (4.4-4.9)	0.568
IVST (cm)	0.9 (0.8-0.9)	0.9 (0.9-1.0)	<0.001
RAD (cm)	3.6 (3.3-3.8)	3.7 (3.4-4.0)	0.185
RVEDD (cm)	3.6 (3.3-3.8)	3.5 (3.3-3.8)	0.777
LVEF (%)	65 (62-68)	65 (62-69)	0.710
MVE (m/s)	1.0 (0.9-1.2)	0.8 (0.7-1.0)	<0.001
MVA (m/s)	0.8 (0.7-0.8)	0.9 (0.8- 1.0)	<0.001
E/A ratio	1.3 (1.2-1.5)	1.0 (0.8-1.2)	<0.001
PH (n/%)	10 (8.5%)	7 (13.7%)	0.298
Mitral regurgitation (n/%)	54 (45.8%)	27 (52.9%)	0.391
Tricuspidregurgitation(n/%)	73 (61.9%)	29 (56.9%)	0.542
Aoric regurgitation (n/%)	16 (13.6%)	16 (31.4%)	0.007

Continuous variables which are non-normally distributed were presented as median (interquartiles); Categorical variables were presented as number (%).

LV: left ventricular; LAD: left atrium diameter; LVEDD: left ventricular end-diastolic diameter; IVST: interventricular septum thickness; RAD: right atrium diameter; RVEDD: right ventricular end-diastolic diameter; LVEF: left ventricular ejection fraction; MVE: peak velocities of the early (E-wave) phase of the mitral inflow pattern; MVA: peak velocities of the late (A-wave) phase of the mitral inflow pattern; PH: pulmonary hypertension.

### Comparison of circulating plasma CTGF levels among hyperthyroidism patients with or without LV diastolic dysfunction

**Figure 1:**
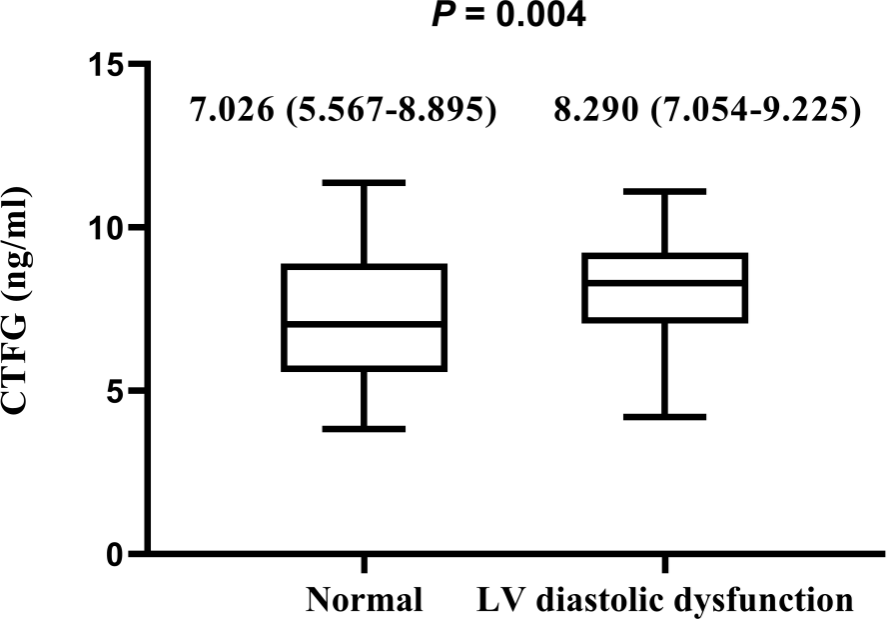
Comparison of circulating plasma CTGF levels among hyperthyroidism patients with or without LV diastolic dysfunction The plasma CTGF levels are presented as median (interquartiles). Differences were analyzed by using the Mann-Whitney U test between two groups. Plasma CTGF levels in LV diastolic dysfunction are significantly higher than those without LV diastolic dysfunction (*P* = 0.004).

Circulating plasma CTGF levels in the LV diastolic dysfunction group were significantly higher than those in the normal group (normal LV diastolic function group: 7.026 [5.567-8.895], LV diastolic dysfunction group: 8.290 [7.054-9.225] ng/ml, median [(Interquartile range)], P = 0.004) (**Figure 1**).

### Correlations of BNP, CTGF, and their ratio with thyroid related parameters and cardiac echocardiographic parameters

Correlations between the serum BNP level, plasma CTGF level, BNP/CTGF ratio and thyroid function-related parameters of the subjects in this study are shown in **Table 3**. The serum BNP level was positively correlated with the serum FT3 and FT4 level (P < 0.001), and Rs were 0.274 and 0.300, respectively. In addition, the serum BNP level was positively correlated with serum TPOAb and TRAb level (P < 0.05), with Rs of 0.155 and 0.153, respectively. However, the plasma CTGF level was not significantly with parameters related to thyroid function. Similar to BNP, we found that the BNP/CTGF ratio was significantly positively correlated with serum FT3 and FT4 levels (P < 0.05). Although the BNP/CTGF ratio was positively correlated with serum TPOAb and TRAb levels, there was not significant. Additionally, we further explored the level of CTGF, BNP, BNP/CTGF according to the duration of disease, but the results were not significant, and the correlation between serum BNP level, plasma CTGF level, and BNP/CTGF ratios with the duration of disease were not significant.

**Table 3:** Correlations of BNP, CTGF and their ratio with thyroid related parameters and the duration of disease.

	BNP		CTGF		BNP/CTGF	
	Rs	*P*-value	Rs	*P*-value	Rs	*P*-value
FT3	0.274	<0.001	0.071	0.359	0.251	0.001
FT4	0.300	<0.001	0.026	0.733	0.289	<0.001
TSH	-0.097	0.210	-0.046	0.552	-0.098	0.204
TPOAb	0.155	0.045	0.043	0.580	0.138	0.074
TgAb	0.103	0.182	0.098	0.205	0.072	0.352
TRAb	0.153	0.047	0.054	0.489	0.131	0.089
Thyroid mass	0.062	0.423	0.010	0.898	0.062	0.423
Duration	-0.029	0.709	-0.025	0.745	-0.012	0.879

TSH, thyroid stimulating hormone; FT4, free thyroxine; FT3, free triiodotronine; TPOAb, anti-thyroid peroxidase antibody; TgAb, anti-thyroglobulin antibody; TRAb, thyrotropin receptor antibody.

The Spearman correlation analysis of serum BNP level, plasma CTGF level, and BNP/CTGF ratios with cardiac echocardiographic parameters are shown in **Table 4**. The serum BNP level was significantly positively correlated with LAD, RAD, MVE, MV and MVA (all P < 0.05), and Rs were 0.339, 0.231, 0.256 and 0.262, respectively. The plasma CTGF level was significantly positively correlated with MVA, with a Rs of 0.179. The BNP/CTGF ratio was significantly positively correlated with LAD, RAD, MVE, and MVA (all P < 0.05), and Rs were 0.305, 0.202, 0.246 and 0.213, respectively.

**Table 4:** Correlations of BNP, CTGF and their ratio with cardiac echocardiographic parameter.

	BNP		CTGF		BNP/CTGF	
	Rs	*P*-value	Rs	*P*-value	Rs	*P*-value
LAD	0.339	<0.001	0.092	0.232	0.305	<0.001
LVEDD	-0.052	0.502	0.092	0.235	-0.078	0.316
IVST	0.081	0.294	-0.049	0.530	0.080	0.302
RAD	0.231	0.003	0.125	0.104	0.202	0.009
RVEDD	0.149	0.052	0.123	0.112	0.118	0.125
LVEF	0.079	0.305	0.004	0.955	0.087	0.262
MVE	0.256	0.001	0.032	0.677	0.246	0.001
MVA	0.262	0.001	0.179	0.020	0.213	0.005
E/A Ratio	-0.017	0.829	-0.069	0.374	0.008	0.917

LAD, left atrium diameter; LVEDD, left ventricular end-diastolic diameter; IVST, interventricular septum thickness; RAD, right atrium diameter; RVEDD, right ventricular end-diastolic diameter; LVEF, left ventricular ejection fraction; MVE, peak velocities of the early (E-wave) phase of the mitral inflow pattern; MVA, peak velocities of the late (A-wave) phase of the mitral inflow pattern; PH, pulmonary hypertension; LV, left ventricular.

### The associations between plasma CTGF levels and LV diastolic dysfunction in the binary logistic regression analysis

As shown in **Table 5**, compared with the bottom quartile group, the crude odds ratios (OR) of LV diastolic dysfunction in the second, third, and fourth quartile group were 3.207, 5.032 and 4.554, respectively in the unadjusted Model 1 (all P<0.05). After adjustment for the potentially confounding variables, such as age, gender, BMI, SBP, DBP, heart rate, and eGFR, the adjusted OR values of the third and fourth quartile group for LV diastolic dysfunction had no obvious change, which were 7.199 and 5.299 respectively, and they were still statistically significant.

**Table 5:** The associations between plasma CTGF level and LV diastolic dysfunction in the binary logistic regression analysis.

Modal	Quintiles of plasma CTGF levels						
	Quartile 1 (0~5.989 ng/ml)	Quartile2 (5.989~7.577 ng/ml)		Quartile3 (7.577~9.104 ng/ml)		Quartile4 (>9.104 ng/ml)	
	OR	OR (95%CI)	*P*-value	OR (95%CI)	*P*-value	OR (95%CI)	*P*-value
Modal 1	1 (Ref)	3.207 (1.027-10.009)	0.045	5.032(1.644-15.404)	0.005	4.554 (1.482-13.991)	0.008
Modal 2	1 (Ref)	3.911 (1.120-13.653)	0.032	6.593 (1.901-22.869)	0.003	4.954 (1.420-17.285)	0.012
Modal 3	1 (Ref)	3.880 (1.112-13.537)	0.033	6.410 (1.844-22.274)	0.003	5.013 (1.438-17.477)	0.011
Modal 4	1 (Ref)	3.408 (0.957-12.128)	0.058	6.800 (1.868-24.748)	0.004	5.303 (1.500-18.749)	0.010
Modal 5	1 (Ref)	3.489 (0.965-12.608)	0.057	6.909 (1.872-25.492)	0.004	5.153 (1.435-18.497)	0.012
Modal 6	1 (Ref)	3.452 (0.950-12.549)	0.060	7.199 (1.927-26.902)	0.003	5.299 (1.461-19.219)	0.011

Modal 1: unadjusted;

Modal 2: age adjusted

Modal 3: age-and sex-adjusted;

Modal 4: adjust for age, sex, and BMI;

Modal 5: adjust for age, sex, BMI, SBP, DBP, and HR;

Modal 6: adjust for age, sex, BMI, SBP, DBP, HR and eGFR.

### ROC for the ability of the serum BNP, plasma CTGF, and BNP/CTGF ratio to differentiate the LV diastolic dysfunction

**Figure 2:**
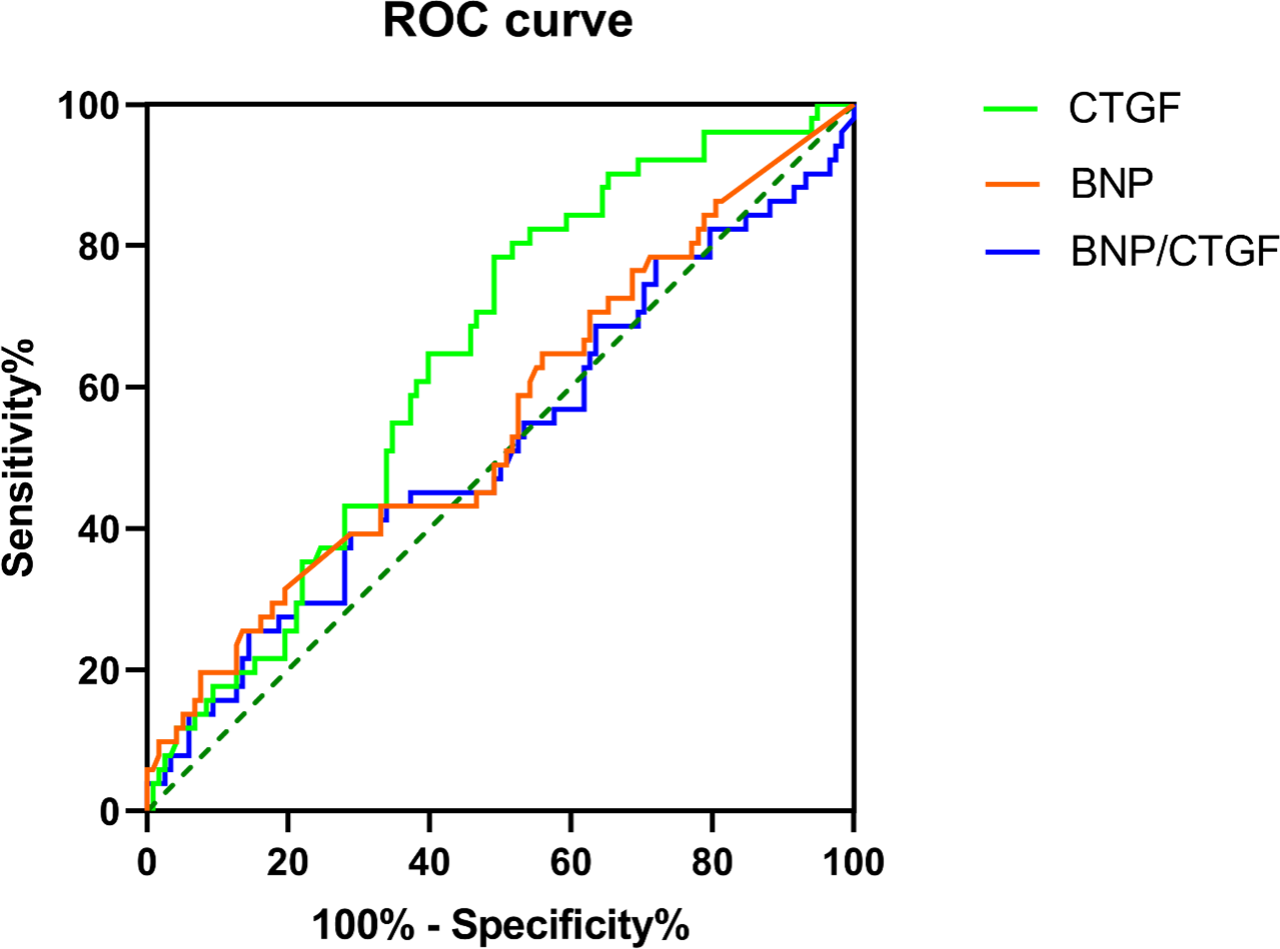
The ROC curve for the ability of the serum BNP level, plasma CTGF level and BNP/CTGF ratio to differentiate the LV diastolic dysfunction.

The ROC of serum BNP, plasma CTGF and BNP/CTGF ratio in predicting diastolic dysfunction are presented in **Figure 2**. The results showed that the plasma CTGF levels had the largest area under the ROC (AUC), and the value was 0.659 (*P* = 0.005), with a sensitivity of 73.0% and specificity of 60.7%. The AUC of serum BNP and BNP/CTGF ratio were 0.588 and 0.547, respectively, but were not statistically significant (P > 0.05).

## Discussion

The results showed that the circulating plasma CTGF level in the LV diastolic dysfunction group was significantly higher than that in subjects with normal LV diastolic function. After adjusting for possible confounding factors (e.g., age, sex, BMI, SBP, DBP, heart rate, and eGFR), plasma CTGF levels remained significantly positively correlated with LV diastolic dysfunction. In addition, we found that serum BNP level and BNP/CTGF ratio were significantly positively correlated with thyroid hormone levels, but no significant correlation between plasma CTGF levels and thyroid hormones was found. Moreover, the serum BNP level and BNP/CTGF ratio were significantly positively correlated with LAD, RAD, MVE, MVA, and plasma CTGF level were significantly positively correlated with MVA. At the same time, The ROC showed that plasma CTGF indicated a significant predictive power in diagnosing LV diastolic function. These results also further confirm that CTGF may be involved in the pathophysiological process of cardiac remodeling.

To our knowledge, this is the first time to explore the association between plasma CTGF levels and LV diastolic dysfunction and the correlation between plasma CTGF levels and thyroid hormone levels in patients with hyperthyroidism. Chi et al. found that plasma CTGF levels were significantly correlated with the degree of LV diastolic dysfunction in patients with diastolic heart failure. The serum BNP level, plasma CTGF level and BNP/CTGF ratio have been found useful in the diagnosis of diastolic heart failure (13). Consistent with these results, we found that plasma CTGF levels remained significantly associated with LV diastolic dysfunction after adjusting for confounding factors in patients with hyperthyroidism, suggesting that plasma CTGF levels are an independent risk factor for LV diastolic dysfunction. However, inconsistently, the ROC showed that plasma CTGF levels were more likely to be used for the diagnosis of LV diastolic dysfunction compared with serum BNP levels and BNP/CTGF ratios, suggesting that measuring plasma CTGF levels is more valuable in the diagnosis of LV diastolic dysfunction in patients with hyperthyroidism, but larger studies are needed. The reason may be that both thyroid hormones and cardiac status can affect the expression of serum BNP, while plasma CTGF levels are less affected by thyroid hormone levels, which is a more realistic reflection of the heart status of hyperthyroid patients than BNP. This was confirmed by our correlation results, where we found a significant positive correlation between serum BNP levels and thyroid hormone levels, which was consistent with previous studies (28, 29). However, we found no significant correlation between plasma CTGF levels and thyroid hormones. Previous studies have supported this to some extent. Previous animal studies have shown that the LV cardiomyocytes of hyperthyroid mice were enlarged in the early stage of hyperthyroidism compared with control, while interstitial collagen fibers were not different or even reduced (32, 33). Additionally, studies have shown that cardiac hypertrophy caused by excessive thyroid hormones initially manifests as physiologic hypertrophy (mainly myocyte hypertrophy, without apoptosis or necrosis of cardiomyocytes and interstitial fibrosis), and normalizes after normal thyroid function (27, 34, 35). All of which suggests that excessive thyroid hormone may not stimulate the activation of profibrotic signaling pathways for a certain period of time or to some extent. In addition, since CTGF is a profibrotic growth factor and BNP is an antifibrotic growth factor, the fibrosis of the ventricles may depend on the balance between the two. Therefore, we speculate that this may be a compensatory mechanism, and excessive thyroid hormones can significantly affect the expression of BNP, while the effect on CTGF is relatively small, so that the CTGF/BNP ratio decreases, thereby inhibiting the excessive production of ventricular collagen fibers and preventing ventricular fibrosis. Additionally, we further explored the level of CTGF, BNP, BNP/CTGF according to the duration of disease, but the results were not significant, and the correlation between serum BNP level, plasma CTGF level, and BNP/CTGF ratios with the duration of disease were not significant. It means we need to include more samples for analysis, as well as explore other possible contributing factors.

Previous studies have shown that CTGF can promote cardiomyocyte hypertrophy and increase extracellular matrix production, leading to myocardial hypertrophy and ultimately heart failure. Chen et al. have shown that CTGF expression levels in atrial tissues are significantly elevated and positively correlated with LAD in patients with atrial fibrillation (36). However, no significant correlation between plasma CTGF levels and LAD was found in this study. The possible reason may be that plasma CTGF levels are not parallel to CTGF levels expressed in myocardial tissue, and patients with concomitant atrial fibrillation were excluded in present study.

This study investigated plasma CTGF levels and further explored the association between plasma CTGF levels and LV diastolic dysfunction and its diagnostic value for LV diastolic dysfunction in clinical hyperthyroidism patients. The cross-sectional observational study design prevented us from determining the causal relationship between LV diastolic dysfunction and plasma CTGF levels. Therefore, our next step will be to further track the plasma CTGF levels and LV diastolic function in these patients after effective therapy. Second, cardiac ultrasound parameters were used to identify LV diastolic dysfunction, which was less accurate than invasive diagnostic methods, and the diagnostic criteria was relatively simple. Also, and we didn’t grade the diastolic dysfunction. In addition, we enrolled only Chinese cohorts, and we are uncertain whether racial differences affected the results. The potential effect of the drug on the expression of plasma CTGF levels was not excluded. Finally, the sample size of this study was relatively small and did not include normal thyroid control groups for comparison.

In summary, we found that the circulating plasma CTGF level in the LV diastolic dysfunction group was significantly higher than those in the normal group in patients with hyperthyroidism, and plasma CTGF level was an independent risk factor for LV diastolic dysfunction. Moreover, compared with serum BNP level, plasma CTGF level may have a more auxiliary diagnostic value for LV diastolic dysfunction.

## Data availability statement

The raw data supporting the conclusions of this article will be made available by the authors, without undue reservation.

## Ethics statement

The studies involving humans were approved by the Committees of Tongji Medical College of Huazhong University of Science and Technology. The studies were conducted in accordance with the local legislation and institutional requirements. The participants provided their written informed consent to participate in this study.

## Author contributions

HL: Data curation, Formal Analysis, Investigation, Methodology, Visualization, Writing – original draft, Writing – review & editing. YR: Methodology, Software, Visualization, Writing – original draft, Writing – review & editing. LW: Project administration, Supervision, Writing – review & editing. YL: Funding acquisition, Project administration, Resources, Supervision, Writing – review & editing.
